# Primary Otomycosis in the Indian Subcontinent: Predisposing Factors, Microbiology, and Classification

**DOI:** 10.1155/2014/636493

**Published:** 2014-05-18

**Authors:** Sampath Chandra Prasad, Subbannayya Kotigadde, Manisha Shekhar, Nikhil Dinaker Thada, Prashanth Prabhu, Tina D' Souza, Kishore Chandra Prasad

**Affiliations:** ^1^Department of Otolaryngology—Head and Neck Surgery, Kasturba Medical College, Mangalore, Manipal University, Karnataka, India; ^2^Department of Microbiology, K.V.G. Medical College, Rajiv Gandhi University of Health Sciences, Sullia, Karnataka, India; ^3^Department of Microbiology, Kasturba Medical College, Mangalore, Manipal University, Karnataka, India

## Abstract

*Objective*. To define otomycosis and determine the predisposing factors and microbiology in primary otomycosis. *Study Design*. Prospective study of two years and review of the literature. *Setting*. Academic Department of Otolaryngology in a coastal city in India. *Patients*. 150 immunocompetent individuals of whom 100 consecutive patients with a clinical diagnosis of otomycosis are considered as the study group and 50 consecutive patients with no otomycosis are considered as the control group. *Results and Observations*. Instillation of coconut oil (42%), use of topical antibiotic eardrops (20%), and compulsive cleaning of external ear with hard objects (32%) appeared to be the main predisposing factors in otomycosis. Aspergilli were the most common isolates (80%) followed by *Penicillium* (8%), *Candida albicans* (4%), *Rhizopus* (1%), and *Chrysosporium* (1%), the last being reported for the first time in otomycosis. Among aspergilli, *A. niger* complex (38%) was the most common followed by *A. fumigatus* complex (27%) and *A. flavus* complex (15%). Bacterial isolates associated with fungi in otomycosis were *S. aureus*, *P. aeruginosa*, and *Proteus* spp. In 42% of healthy external ears fungi were isolated. *Conclusion*. *Aspergillus* spp. were the most common fungi isolated, followed by *Penicillium*. Otomycotic ears are often associated with bacterial isolates when compared to normal ears. Fungi are also present in a significant number of healthy external auditory canals and their profiles match those in cases of otomycosis. The use of terms “primary” and “secondary” otomycosis is important to standardize reporting.

## 1. Introduction

Otomycosis or fungal otitis externa has typically been described as fungal infection of the external auditory canal with infrequent complications involving the middle ear. Although rarely life threatening, the disease is a challenging and frustrating entity for both patients and otolaryngologists as it frequently requires long-term treatment and follow-up. Despite this, there could be recurrences. Otomycosis is one of the common conditions encountered in a general otolaryngology clinic setting and its prevalence has been quoted to range from 9% [1] to 27.2% [2, 3] among patients who present with signs and symptoms of otitis externa and up to 30% [4–6] in patients with discharging ears. It is worldwide in distribution with a higher prevalence in the hot, humid, and dusty areas of the tropics and subtropics [2, 3, 5, 6]. Overview of the literature reveals otomycosis to be a common medical problem in India [7, 8]. Fungi can either be the primary pathogen or be superimposed on bacterial infections. Most patients suffering from early otomycosis complain of severe itching which often progress to pain, hearing loss, and often leading to tympanic membrane perforations [8–10]. Although* Aspergillus niger* and* Candida albicans* are by far the most common offenders [2, 5–8, 10], a wide spectrum of other fungi can cause otomycosis. Various factors have been proposed as predisposing factors for otomycosis, including a humid climate, presence of cerumen, instrumentation of the ear, immunocompromised host, and, more recently, increased use of topical antibiotic/steroid preparations.

In this study conducted in a rainy and humid coastal city in South India, we identify the organisms isolated in otomycosis, including fungi and bacteria and compare them with normal ears.

## 2. Materials and Methods

A total of 150 immunocompetent individuals attending the Outpatient Department of Otolaryngology—Head and Neck Surgery at Kasturba Medical College in Mangalore, a coastal city in the west coast of India, were chosen for the study. This was performed over a two-year period between January 2008 and January 2010.

*Inclusion Criteria.* 100 consecutive patients with clinically diagnosed otomycosis in the absence of other ear conditions like chronic otitis media were included in the study group. Such patients were considered to have primary otosclerosis. Further 50 consecutive outpatients without clinical otomycosis or any aural symptoms, seeking treatment for minor nonotological ailments with no bearing on otomycosis, were included in the control group. The criteria used for establishing a diagnosis of otomycosis were based on* any two or more* of the following findings:

1. presence of symptoms like itching, pain, feeling of blocked ear, tinnitus, deafness, and otoscopy revealing masses of hyphae/spores or a curd-like grey/white discharge,
2. the demonstration of fungal elements in 10% potassium hydroxide-methylene blue preparation,
3. culture of clinical material (debris, discharge, and moulds) on Sabouraud's dextrose agar slants.

*Exclusion Criteria*. The following conditions were classified as secondary otomycosis and were excluded from the study:

(1) all patients with otomycosis alongside history of chronic otitis media, active otitis, tympanic membrane perforations, prior ear surgery or aural procedures, and diabetics,
(2) patients with any serious debilitating diseases like malignancies, tuberculosis, and other chronic granulomatous diseases,
(3) patients with immunocompromised conditions and history suggestive of fungal diseases elsewhere in the body.

Clinical presentations of cases such as itching, pain, feeling of ear blockage, and ear discharge were recorded. Any history of trauma, use of wooden sticks, metal wax pickers or any other objects in an attempt to remove ears wax from ear, use of oils, topical antibiotic ear drops, and/or other aural preparations were noted. Age, sex, socioeconomic status, and occupation of the patient were also recorded. A history indicative of fungal infections elsewhere in the body like onychomycosis and recurrent vaginal infections (in females) was elicited to rule out otomycosis secondary to seeding of infections. Patients' nails were checked for any evidence of onychomycosis. In patients with otomycosis, scrapped material was collected from the external auditory canal with the help of a sterile Jobson Horne probe with ring curette and cotton carrier. In all other cases, specimens were collected using three sterile cotton tipped swabs and transported to the laboratory within half an hour for mycological and bacteriological examination. Routine blood and urine analysis, ELISA for HIV, and blood sugar tests for diabetes (diabetes was determined by fasting blood sugar result above 100 milligrams of glucose per deciliter of blood) were done in all cases to rule out immunocompromised states among the study and control populations. Consent was taken from all patients involved in the study.

*Processing.* A portion of the scrapped material or one of the swabs was cultured on blood and MacConkey's agar at 37°C for 24 h and 48 h and examined for bacterial growth. Identification of the bacterial isolate was done by standard procedures [11]. Second swab/scrapped material was digested on a microscopic slide with 10% potassium hydroxide-methylene blue (2 : 1) [11]. A 22 mm square number 1 cover slip was placed on the digested material and examined microscopically using 10 X and 40 X objectives for fungal elements. The third swab/scrapped material was inoculated on two Sabouraud's dextrose agar with chloramphenicol. One of the agar slants was incubated at room temperature (25°C) and the other was incubated at 37°C for 2 to 3 weeks. Cultures were examined for growth on alternate days. Fungi were identified by standard procedures [12].

All patients diagnosed with otomycosis were subjected to a thorough aural toilet by suctioning and removal of the fungal debris. Following this patients were prescribed clotrimazole antifungal preparations, 4-5 drops to be instilled 3 times a day for a minimum of 7 days and a maximum of 14 days. In case of associated coexisting otitis externa, a combined antifungal-antibiotic preparation was prescribed for the same period. Ear swabs collected from the control group were also processed by similar methods as for otomycosis patients.

Data was analysed with a statistical software program (SPSS Statistics for Windows version 20, Chicago, IL). Categorical data was presented as frequencies and percentages. The association between the predisposing factors (self-cleaning, eardrops, and oil instillation) and the prevalence of otomycosis was analyzed with the chi-square test. All *P* values were calculated with one tail. *P* values below 0.05 were considered significant.

## 3. Results and Observations

### 3.1. Age, Sex, Occupation, and Socioeconomic Status

**Figure 1:**
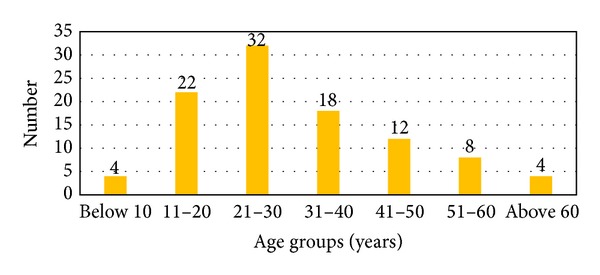
Age distribution of subjects in the study (otomycosis) group.

63 (63%) of the patients with otomycosis (study group) were males and 37 (37%) were females (Table 1). In the control group 30 (60%) were males and 20 (40%) were females. The highest incidence of otomycosis was in the age group of 21–30 years and the lowest was noted in the age group of less than 10 years and over 60 years of age (Figure 1). The highest incidence of isolation of fungi in the control group was in the age groups of 21 to 40 years. Agriculture was the main occupation in 105 (70%) of patients. 60 (40%) of the males and 45 (30%) of the females were engaged in agriculture related jobs in a day-to-day basis. 94 (63%) of the patients had an average family income of INR (Indian National Rupees) 1050 to 5000 per month and 56 (37%) had an average family income of INR 20,000 per month and above.

### 3.2. Side and Laterality

Otomycosis was predominantly unilateral both in males and in females. Only 5% presented bilateral infection. In the females, the right ear was most commonly involved (23/37 cases, 62%) whereas in males the distribution was almost equal in both ears (right ear 49% and left ear 46%). Overall right ear was involved in 54% of the study group. 95% of the subjects with otomycosis were right handed. No such association was observed in control group.

### 3.3. Predisposing Factors

**Figure 2:**
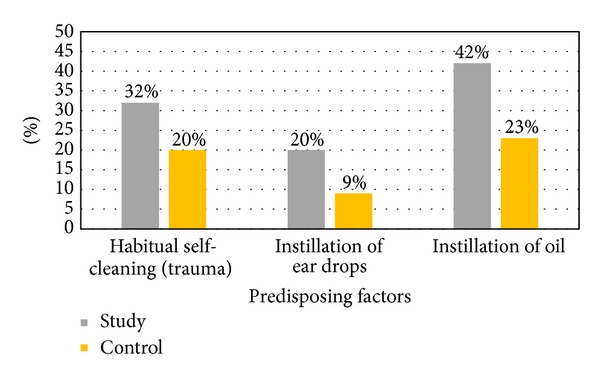
Comparison of predisposing factors between the study (otomycosis) and the control groups.

The incidence of otomycosis was high in patients with history of instilling coconut oil (42%) into the ear. In the otomycosis group, history of habitual cleaning of the ear by the patient with unsterile sticks, metal wax picks, or rolled bus tickets was recorded in 32% of the cases; the use of topical antibiotics eardrops in 20% and the habit of instilling oil in the ear was recorded in 42% of patients. In the control group, history of habitual cleaning of the ear, use of ear drops, or instillation of oil was also observed in 20%, 9%, and 29% of the subjects, respectively (Figure 2). The prevalence of history of self-cleaning, use of eardrops, or oil instillation (32%, 20%, and 42%, resp.) was significantly higher in the otomycosis group when compared to the control group (20%, 9%, and 29%). These three factors were statistically associated to a higher incidence of otomycosis (*P* = 0.0265, *P* = 0.0136, and *P* = 0.0274, resp.).

### 3.4. Clinical Features

Itching was the predominant symptom seen in 73% of the otomycotic patients followed by a blocked sensation in the ears (38%). Other symptoms were ear discharge (38%), ear pain (35%), and tinnitus (8%).

### 3.5. Microbiology

Fungi were isolated from all 100 patients constituting the study group who were diagnosed clinically to have otomycosis (Table 2). Species of* Aspergillus* were isolated in 80% cases.* A. niger* complex was the commonest (38%) followed by* A. fumigatus* complex (27%) and* A. flavus* complex (15%).* Penicillium* species (8%),* Candida albicans *(4%),* Rhizopus *spp. (1%), and* Chrysosporium *spp. (1%) were the other fungi isolated. Single fungus was isolated from 94% of cases. Two fungi each were isolated from six cases. Of these six cases, three had coexisting* A. niger* complex and* A. fumigatus *complex and three had coexisting* A. niger* complex and* A. flavus *complex. Fungi were isolated from 42% of the healthy external auditory canals (controls). The fungi isolated from healthy ears were predominantly aspergilli (30%).* A. niger* complex was the commonest (22%) followed by* A. fumigatus *complex(4%) and* A. flavus *complex (4%).* Candida albicans* (6%),* Penicillium *spp. (4%), and* Rhizopus *spp. (2%) were the other species isolated. 63% of patients with otomycosis had associated bacterial infections (Table 2). In the study group, the commonest bacterial association observed was coagulase positive staphylococci (31%) followed by* Pseudomonas aeruginosa* (20%),* Proteus *spp. (7%), and* Klebsiella *spp. (5%). In the control group,* S. aureus* 1 (9%) and* S. epidermidis* (37%) were the most common organisms isolated.

### 3.6. Treatment

23% of the otomycotic patients were treated with clotrimazole alone and were cured completely. 77% were treated with combined antifungal and antibiotic preparations and were cured completely of the disease.

## 4. Discussion

Andrall and Gaverret were the first to describe fungal infections of the ear [7, 13]. Otomycosis is a superficial mycotic infection of the outer ear canal. The infection may be either subacute or acute and is characterized by inflammation, pruritus, scaling, and severe discomfort. The mycosis results in inflammation, superficial epithelial exfoliation, masses of debris containing hyphae, suppuration, and pain.

### 4.1. Geography

The incidence of otomycosis is reported to be high in tropical countries [2, 3, 5, 6, 14]. Mangalore, the city where the present study has been done, is a coastal city in South India where the ambient temperature varies from a minimum 17°C to a maximum 38°C. There is a heavy rainfall of about 4000 mm per annum of which about 90% was received in the monsoon months between June and September. The relative humidity is generally very high reaching saturation levels during the monsoon months. Warmth and moisture are highly conducive for the growth of fungi. The incidence of otomycosis was high (78%) in the rainy months of July, August, and September compared to the months between January and March (22%).

### 4.2. Age, Sex, and Occupation

While some studies have reported otomycosis to be more common among young men [1, 5, 7, 8, 15] as in our series, many others have reported it to be so among young females [2, 3, 14, 16, 17]. In our series the male : female ratio was 1.7 : 1. In our series, patients with otomycosis mainly came from an agricultural background. In a study by Wadhwani and Srivastava [13], 24 fungi were isolated from earwax or otitis media of agricultural field workers, of which 18 were being reported for the first time from India. Young men generally spend more time outdoors and aspergilli are common airborne saprophytes. Previous reports have stated an increased incidence in people of lower socioeconomic status [7, 14, 16] and this was also seen in our series.

### 4.3. Predisposing Factors

The skin covering the external auditory canal is similar to the other parts of the body, but it is exposed to the atmosphere by a small meatal inlet. In other words, the anatomical disposition of external auditory canal simulates a culture tube lined with skin that provides ideal condition for fungal and bacterial growth [15]. Further anatomical aberrations like a narrowing of the canal may also predispose to otomycosis. The presence of excessive cerumen in patients with poor personal hygiene favors the germination of spores and conidia of the prevalent fungi [7]. Obsessive manipulation of the external ear with any hard objects such as wooden sticks or metal wax picks to clean the ear of wax and vigorous rubbing of the ears for relief from itching (in case of otalgia due to eustachian catarrh, serous otitis, or acute otitis media) is a common practice. These practices may cause trauma (usually minor and hence unnoticed) to the skin of external auditory canal and deposition of fungal conidia in the wound leading to fungal infection [17]. The moisture, warmth, and acidic pH of the external auditory canal provide ideal growth requirements for the fungi. Aspergilli have an optimum pH range of 5.7 and a maximum growth rate at a temperature of 37°C and this is conducive for all species of* Aspergillus* isolated in the present study. This is supported by the predilection of fungi to grow in the inner one-third of external auditory canal. Swimming is also indicated as a predisposing factor for otomycosis [2, 5, 16]. Onychomycosis and other forms of dermatomycoses are a potential source of repeated autoinoculation [18, 19]. If the nails are thickened, white, and crumbling, they should be trimmed and sent for culture and systemic therapy should be considered. Some women with recurrent vaginal candidiasis also develop candidal otomycosis. The flare-ups in these cases are hormonally related and also require systemic therapy [20].

Lack of formal education in people in many parts of India has led them to believe myths that coconut oil application for ears is beneficial for a variety of ear ailments. Our study revealed high association (42%) of otomycosis with instillation of coconut oil into the external ear. Coconut oil has been reported to be sporostatic [21] and therefore may help preserve the viability of fungal conidia deposited in the external ear long and indirectly contribute to occurrence of otomycosis. Similarly the use of mustard oil is associated with high incidence of otomycosis [5].

In a series by Oliveri and coworkers [22] lack of cerumen, chronic otitis, previous antibiotic therapy, and eczema were common predisposing factors. They also mention that working in gardens or using mechanical removing devices was significant predisposing factors. Cerumen, whose role is protective in the external auditory canal, was absent in majority (93%) of the patients in our series. Prior treatment of a bacterial infection with long-term topical antibiotic therapy is indicated as a predisposing factor presumably due to the protective cerumen layer [6, 23, 24]. 20% of the patients in our series used over-the-counter antibiotic eardrops for itching and pain in the ear without medical consultation.

### 4.4. Signs and Symptoms

Symptoms of otomycosis include pruritis, pain, otorrhoea, and hearing loss. Clinical appearance of aspergillosis and candidiasis of the ear canal differs considerably. Aspergillosis is characterized by a mild moist inflammation of the deep ear canal. The lumen is filled with large shed sheets of keratin that have a wet tissue-paper appearance.* Candida* usually causes greater edema and maceration of the deep ear canal. The lumen may be filled with a curd-like material [20]. Otomycosis can also lead to tympanic membrane perforations [5, 9, 17, 25] and spread to the middle ear. For documentation purposes and standardization, it is essential for the treating otolaryngologist to define otomycosis as primary or secondary. We propose the following classification.

- Primary otomycosis. Otomycosis present in the external auditory canal in an immunocompetent individual and in the presence of an intact tympanic membrane and absence of any other external or middle ear pathology.
  - Without otitis externa. Clear absence of clinical signs of otitis externa at the time of presentation like inflamed canal wall or stenosis of the auditory canal.
  - With otitis externa. Presence of clinical signs of otitis externa, when no clinical manifestation of otitis externa was present prior to the time of presentation. That is, otitis externa was due to otomycosis and not vice versa.
- Secondary otomycosis. Otomycosis present in the EAC or middle ear alongside history of and/or existing otitis media or externa, trauma, or postoperative ears or with history of and/or existing fungal infections in other parts of the body.
  - Without immunocompromise. No HIV, diabetes mellitus, or granulomatous diseases or other immunocompromised conditions detected by lab studies.
  - With immunocompromise. Presence of any immunocompromised condition detected by lab studies.

### 4.5. Investigations and Organisms Involved

Simple culture and microscopic examinations under sterile conditions are sufficient to confirm otomycosis.* Aspergillus* is the most commonly reported isolate in otomycosis across the world, followed by* Candida* [2, 5–7, 10, 13–15, 22, 26]. This is not different from reports from the Indian subcontinent, including our study [5, 7, 8, 16, 27–30]. In a few series the incidence of* Candida* has been reported to be higher than* Aspergillus* [2, 4, 31]. However, in our study,* Penicillium* (8%) was the second most common isolate after* Aspergillus* with* Candida albicans* constituting only 4%. The involvement of* Penicillium *spp. in our series was also found to be higher compared to earlier reports from India and other parts of the world [7, 8, 16, 32–34]. The isolation of* Chrysosporium *spp. in our series is the first of its kind and its pathogenic role in otomycosis yet to be elucidated.* Chrysosporium *spp. have occasionally been isolated from systemic infections in bone marrow transplant recipients and in patients with chronic granulomatous diseases. However our patient had no such associated factors. Fungal isolates in otomycosis from reports across the world are compared in Table 3.

Apart from otomycosis,aspergilli cause a wide spectrum of infections including cutaneous manifestations and invasive infections such as pulmonary aspergillosis and endocarditis. Clinical microbiology laboratories rely heavily on morphology-based identification methods for* Aspergillus* species wherein diagnostic criteria include the recognition of asexual or sexual structures and their characteristics such as shape, size, color, ornamentation, and/or mode of attachment. Unfortunately, such a phenotype-based scheme is not effective in identifying the species because largely these characteristics are unstable, and clinical aspergilli sometimes manifest atypically with slow sporulation and aberrant conidiophore formation [35]. Additionally, members of the section* A. Fumigatus* have overlapping morphological characteristics, with several genetically distinct species existing within a single morphospecies. Clinically, it is important to identify* Aspergillus* species because different species have variable susceptibilities to multiple antifungal drugs thereby influencing the choice of appropriate antifungal therapy. For example, studies have demonstrated that* A. terreus* isolates are largely resistant to the antifungal drug amphotericin B. Comparative DNA sequence-based identification formats appear to be promising in terms of speed, ease, objectivity, and economy for species identification [35]. Not using molecular methods to ascertain the species could be a drawback in our study. However, in spite of the shift of fungal identification formats into the molecular arena there is no consensus on the gene/genes that can be used for species identification in the genus* Aspergillus* [35]. Also, this methodology involves significant cost and phylogenetic expertise that are limiting factors in most clinical microbiology laboratories. Additionally, consideration should also be given to the fact that most of these isolates may not be true causative agents of disease and therefore may not warrant species level identification in a diagnostic laboratory. Taken together, a universal single marker that would rapidly and accurately identify* Aspergillus* isolates to the species level would help support diagnostic microbiology laboratories in their routine identification efforts [35].

*Aspergillus niger* complex is one of the most common species which is also a oligotroph; that is, they are capable of growing even in nutrient-depleted environments. This is also one of the important microorganisms used in biotechnology. It has been in use already for many decades to produce extracellular (food) enzymes and citric acid. In addition,* A. niger* complex is used for biotransformations and waste treatment. In the last two decades,* A. niger* complex has been developed as an important transformation host to overexpress food enzymes.* A. niger* complex, like other filamentous fungi, should be treated carefully to avoid the formation of spore dust [36]. This spore dust could lead to contamination of external auditory canals predisposing to otomycosis. The human external auditory canal is an ideal environment for this fungus to grow and abundance of proteins and carbohydrates and favorable humidity and temperature explain this finding [14, 33]. On the other hand,* Candida* possess constitutive hydrolytic enzymes to aid invasion of host tissues and in this investigation the majority of tested* Candida* have been demonstrated to have protease activity. These findings suggest that protease production may play an important role in the pathogenesis of otomycosis caused by* Candida*. It is possible that protease enzymes enhance the ability of* Candida* to colonize the skin and penetrate host cells, which could be important in establishing the infection in the ear [37]. Also, despite the fact that many authors did not clearly differentiate between primary otomycosis and otomycosis secondary to otitis media,* Candida* was found more commonly when compared to* Aspergillus* in postoperated cavities or infected middle ears [4, 9, 38] and in immunocompromised individuals [8, 39].* Aspergillus* is considered a primary colonizer of the ear canal [17, 27].

Like skin elsewhere in the body, that of the external auditory meatus has a normal commensal flora such as* Staphylococcus epidermidis* (*albus*) and* Corynebacterium *spp. In addition,* Staphylococcus aureus* and* Streptococcus viridans* can frequently be present without causing ill effects. When the skin's natural defense mechanisms break down, as in otitis externa, the resident bacteria multiply because of the more favorable environment and other organisms such as* Proteus* and* Pseudomonas *spp., which are normal commensals of other parts of the body, may then flourish [40]. In our series,* Staphylococcus aureus, P. aeruginosa,* and* Proteus *spp. were dominant bacterial pathogens which is in line with previous reports [1, 32, 33].* A. fumigatus* was the only one of the aspergilliwhich was not accompanied routinely by* S. aureus* which is also in line with previous reports [1]. This has been attributed to an antibiotic activity against* S. aureus *formed by* A. fumigatus* [1].

In the control group, fungi were seen in 42% of the normal ears. The order of frequency of isolation of fungi from healthy external ear was almost similar to that observed in cases of otomycosis and the isolation rate of aspergilli from healthy external ears was significant (30%).* A. niger *complex*, A. fumigatus *complex*, A. flavus *complex*, C. albicans, Penicillium *sp*., and Rhizopus *were isolated. This leads us to the question: do most normal ear canals harbor fungal commensals? The answer to this could be in the negative as a majority of the normal ears (58%) did not show any fungus. However, it can be reasonably opined that the presence of fungus in normal ears could be due to a decreased host barrier in the canal skin due to either minor trauma (that may go unnoticed) during ear cleaning, itching (it is usual for patients to itch the ear in case of otalgia due to eustachian catarrh, serous otitis, or acute otitis media), or lacerations due to accidents. Factors like poor sanitation, poor nutrition, immunocompromised states (human immunodeficiency virus infection and diabetes mellitus), aberrant anatomy of the canal, or physical factors like increased regional humidity or temperatures may also tilt the balance towards growth of fungus. Such patients with fungus in the ears without symptoms or signs can be considered to be “predisposed” to the condition and may develop frank otomycosis in a course of time if the favoring conditions persist.

### 4.6. Treatment

Treatment involves elimination of predisposing factors. Topical antibiotic solutions must be stopped. Patients' nails must be inspected to rule out onychomycosis. The ear canal must be thoroughly debrided of all visible debris. It is our practice to avoid syringing and clear the debris by suctioning alone. Fungicidal drops are the most popular form of treatment [26, 41]. Clotrimazole has an antibacterial effect, and this is an added advantage when treating mixed bacterial-fungal infections [6]. Fungicidal creams with ketoconazole or fluconazole may also be applied [6, 41]. A readily available and usually effective preparation for* Candida* is tolnaftate, available over the counter for the treatment of athlete's foot. Nystatin is another useful drug against* Candida*. The major advantage of nystatin is the fact that it is not absorbed across intact skin. Nystatin is not available as an otic preparation; however it can be prepared as a solution or a suspension for the treatment of otomycosis [6]. Studies on animals with experimentally induced fungal infections have furnished evidence for the risk of the infections' spreading to the inner ear and causing serious damage to the organ of Corti; indirect damage to these structures by mycotoxins cannot be ruled out. In rare refractory cases of otomycosis due to HIV or other immunocompromised states, or in life threatening condition, parenteral antifungals like amphotericin B or tolnaftate may be used [6, 39].

## 5. Conclusion

Otomycosis is seen across the world with a high incidence especially in tropical countries. In this study we analyzed the growth of fungi and bacteria in 100 otomycotic ears and compared it with 50 healthy ears. In a study conducted in a coastal Indian city,* Aspergillus* spp. were the most common fungi isolated. This was followed by* Penicillium* which is uncommon in other reports. The isolation of* Chrysosporium* has not been reported before in otomycosis. Otomycotic ears are often associated with bacterial isolates when compared to normal ears. Fungi are also present in a significant number of healthy external auditory canals and their profiles match those in cases of otomycosis. The use of terms “primary” and “secondary” otomycosis is important to standardize reporting.

## Figures and Tables

**Table 1:** Demographic profile of the subjects in the study (otomycosis) and control groups.

Age	Sex	Study group (otomycosis)	Control group
<10 years	Male	3	—
	Female	1	—

11–20 years	Male	13	7
	Female	9	3

21–30 years	Male	20	12
	Female	12	5

31–40 years	Male	12	8
	Female	6	3

41–50 years	Male	8	5
	Female	4	2

51–60 years	Male	5	3
	Female	3	1

>60 years	Male	2	1
	Female	2	—

Total	Male	**63**	**36**
	Female	**37**	**14**

**Table 2:** Fungi-bacteria association in study (otomycosis) and control groups.

Fungal isolates	Study group (*n* = 100)		Control group (*n* = 50)		Study group (*n* = 100)	Control group (*n* = 50)
	Number of cases positive for fungi (%)	Number of cases positive for bacteria (%)	Number of cases positive for fungi (%)	Number of cases positive for bacteria (%)	Associated bacterial isolates, number (%)	
*A. niger *complex	38 (38)	25 (66)	11 (22)	5 (13.2)	*S. aureus* 12 (31.5) *Proteu*s 3 (7.9) *P. aeruginosa* 7 (18.4) *Klebsiella* 3 (7.9)	*S. aureus* 1 (9.1) *S. epidermidis* 4 (36.4)

*A. fumigatus *complex	27 (27)	17 (63)	2 (4)	1 (3.7)	*S. aureus* 4 (14.8) *Proteus* 3 (11.1) *P. aeruginosa* 8 (29.6) *Klebsiella* 3 (11.1)	Corynebacteria 1 (50)

*A. flavus *complex	15 (15)	9 (60)	2 (4)	0 (0)	*S. aureus* 7 (14.8) *Proteus* 1 (3.7) *P. aeruginosa* 1 (3.7)	0 (0)

*Penicillium *spp.	8 (8)	2 (25)	2 (4)	0 (0)	*S. aureus* 2 (25)	0 (0)

*C. albicans *	4 (4)	3 (75)	3 (6)	0 (0)	*S. aureus* 1 (25) *P. aeruginosa* 2 (50)	0 (0)

*Rhizopus *spp.	1 (1)	1 (100)	1 (2)	0 (0)	*S. aureu*s 1 (100)	0 (0)

*Chrysosporium *	1 (1)	1 (100)	0 (0)	0 (0)	*S. aureus* 1 (100)	0 (0)

Mixed fungal infections	6 (6)	5 (83)	0 (0)	0 (0)	*S. aureus* 3 (50) *P. aeruginosa* 2 (38.3)	0 (0)

Total	**100 (100)**	**63 (63)**	**21 (42)**	**6 (12)**		

**Table 3:** Percentage of various fungi in otomycosis as reported by different workers over the last few decades.

	Reports from the Indian subcontinent							Reports from other parts of the world							
Authors	Joy et al. [32]	Jain and Agrawal [21]	Jaiswal [42]	Kaur et al. [7]	Pradhan et al. [5]	Viswanatha et al. [8]	Present study	Geaney [43] UK	Yassin et al. [44] S. Arabia	Pontes et al. [2] Bahrain	Yehia et al. [15] Iraq	Fasunla et al. [3] Nigeria	Pontes et al. [2] Brazil	Jia et al. [17] China	Barati et al. [14] Iran

Year	1980	1992	1990	2000	2003	2012	2014	1967	1978	2009	1990	2008	2009	2012	2011

*Asp. niger *	44.3	56.3	34	36.9	25.5	56	38	13.2	51.2	54.4	70.1	48.35	20	54.77	41.6
*Asp. fumigatus *	15.7	15.6	—	41.1	6.6	18	27	7.5	—	25.1	5.6	33.96	5	2.61	5.5
*Asp. flavus *	23.2	4.7	—	1.4	37.7	—	14	9.2	18.3	—	15.6	5.43	10	6.09	49

Other aspergilli	—	3.1	—	—	0.9	—	—	34.9	13.7	—	—	—	—	9.57	3.7

*Candida* sp.	7.6	6.3	46	13.7	10.4	16	4	35.2	4.6	17	7.3	12.26	55	24.35	7.6

*Mucor* sp.	5.4	6.3	—	1.4	—	—	—	—	2.3	—	—	—	—	—	—

*Penicillium* sp.	1.1	4.7	—	1.4	—	10	8	—	5.3	3.5	—	—	—	—	—

*Rhizopus *	—	—	12	2.7	—	—	1	—	—	—	0.6	—	—	—	—

*Chrysosporium *	—	—	—	—	—	—	1	—	—	—	—	—	—	—	—

Other fungi/mixed fungi	2.7	3	8	1.4	—	—	6	—	4.6	—	—	—	10	2.61	0.9

No growth	—	—	—	—	18.9	—	—	—	—	—	—	—	—	—	—
